# Feasibility and Implementation Considerations for Long-Acting Antiretroviral Therapy in Pakistan: A Qualitative Study of Healthcare Providers and Programmatic Stakeholders

**DOI:** 10.1177/23259582261452317

**Published:** 2026-05-19

**Authors:** Muhammad Wasay Shahid, Masood-ur-Rehman, Faizur Rehman, Mehran Riaz, Muhammad Aaqib Akram, Farah Azhar, Malik Muhammad Umair, Nasim Akhtar, Musarat Jabeen, Ali Ahmed

**Affiliations:** 1 Riphah Institute of Pharmaceutical Sciences, 66783 Riphah International University, Islamabad, Pakistan; 2 66971 Al-Hamad Pharmacy, Islamabad, Pakistan; 3 66783 National AIDS Control Program, Common Management Unit, Islamabad, Pakistan; 4 Department of Infectious Diseases, 66688 Pakistan Institute of Medical Sciences, Islamabad, Pakistan; 5 Antiretroviral Therapy Centre, 66688 Pakistan Institute of Medical Sciences, Islamabad, Pakistan; 6 Division of Infectious Diseases and Global Public Health, 21814 School of Medicine, University of California San Diego (UCSD), La Jolla, California, USA; 7 Perelman School of Medicine, University of Pennsylvania, Philadelphia, PA, USA

**Keywords:** long-acting injectables. LAI-ART, cabotegravir-rilpivirine, LA-CAB/RPV, Pakistan, HIV/AIDS, antiretroviral therapy, people living with HIV, PLHIV, feasibility and acceptability

## Abstract

**Introduction:**

Long-acting injectable antiretroviral therapy (LAI-ART), especially cabotegravir plus rilpivirine, is primarily recommended as a switch option for virally suppressed individuals and may improve adherence, privacy, and treatment satisfaction. Contemporary international guidance notes a restricted, closely monitored role for selected viraemic patients with persistent adherence challenges. Feasibility in low-resource systems remains uncertain. We explored perceived feasibility and acceptability of LAI-ART among HIV care providers and regulatory stakeholders in Pakistan.

**Methods:**

We conducted semistructured in-depth interviews with 17 providers and stakeholders from ART centers, tertiary hospitals, the National AIDS Control Program, and a community-based organization in Islamabad and Rawalpindi. Interviews were audio recorded, transcribed, translated, and analyzed using reflexive thematic analysis.

**Results:**

Participants viewed LAI-ART as promising for improving adherence, treatment fatigue, confidentiality, and workflow. Barriers included travel distance, needle phobia, missed dose risks, staffing constraints, cold chain and storage needs, infrastructure limitations, and weak biomedical waste management.

**Conclusion:**

Stakeholders expressed cautious interest in LAI-ART while highlighting significant structural, financial, and sociocultural barriers. Participants emphasized that phased pilot implementation, sustainable financing, cultural acceptability, integration into differentiated service delivery, and stronger counselling and digital support would be necessary to support future introduction.

## Plain Language Summary Title

Are Long-Acting HIV Injections Feasible and Acceptable in Pakistan? A Qualitative Study

**Plain Language Summary:**

This study explored whether long acting HIV injections could be introduced in Pakistan and whether they would be acceptable to those involved in HIV care and regulation. We interviewed 17 key informants, including physicians, pharmacists, counsellors, nurses, and regulatory stakeholders. Participants viewed long acting injectable treatment positively and believed it could improve adherence, privacy, and treatment satisfaction. However, they identified important barriers, including long travel distances, needle phobia, missed dose risks, staffing shortages, cold chain and storage needs, infrastructure limitations, and biomedical waste management gaps. They recommended phased pilots, strong financing, culturally acceptable delivery models, and better counselling and digital support before wider rollout.

## Introduction

Global HIV treatment has been significantly transformed by long-acting injectable antiretroviral therapy (LAI-ART), particularly the monthly or bi-monthly cabotegravir and rilpivirine (LA-CAB/RPV) regimen, which replaces daily oral dosing with infrequent intramuscular injections.^1,2^ As an alternative to standard oral ART, the injectable formulation confers notable clinical and psychosocial benefits by mitigating adherence challenges, adverse effects (AEs), drug interactions, pill burden, travel burden and associated stigma, thus improving quality of life.^3,4^ Accumulating evidence from clinical trials reports that LA-CAB/RPV achieves viral suppression comparable to that of standard oral ART.^5–7^

In response to the favorable clinical outcomes, LA-CAB/RPV has been incorporated into HIV treatment programs across diverse economic settings.^8^ Increasing research has focused on evaluating acceptability of LAI-ART across various settings. Evidence from both high-income countries (HICs)^9,10^ and low-resource settings^11,12^ indicates that HIV care providers generally regard LAI-ART as a promising and acceptable alternative to daily oral therapy, particularly for individuals struggling with adherence or stigma. Providers in HICs express optimism about its potential yet acknowledge barriers such as increased clinic workload, staffing demands, logistical challenges (eg, cold chain storage, procurement), and financial or insurance constraints. These factors often restrict feasibility to people living with HIV (PLHIV) who are stable with sufficient resources.^13–15^ Similarly, in low- and middle-income countries (LMICs), healthcare providers recognize the benefits of LAI-ART but cite comparable challenges, including limited infrastructure, regulatory delays, supply chain inefficiencies, and the need for specialized training.^11,16,17^

In Pakistan, the HIV epidemic continues to challenge national treatment and prevention goals. The care continuum from diagnosis to treatment is constrained by significant system level challenges. Although concentrated among key populations, the epidemic has gradually spread into the general population,^18^ with an estimated 330,000 PLHIV in Pakistan.^19^ Pakistan has developed a growing ART infrastructure to address this need, however challenges persist,^20^ with only 58,622 of 81,847 diagnosed individuals receiving treatment.^19^ According to the Joint United Nations Programme on HIV/AIDS (UNAIDS), merely 23,000 ART users (39%) achieved viral suppression in 2024,^21^ a substantial decline from 75% reported in 2023.^22^ This widening treatment gap deviates from the UNAIDS 95-95-95 targets and reflects persistent weakness in HIV care coverage and viral suppression.

Poor adherence rates to oral ART in Pakistan stem from multiple intersecting factors, including HIV-related stigma, mental health challenges,^23^ pill fatigue, AEs, economic hardship, and logistical barriers especially during COVID-19 pandemic.^24^ LAI-ART offers a potential alternative approach to address these barriers by improving adherence, reducing dosing frequency, and enhancing confidentiality among individuals who are already engaged in care and virally suppressed on oral ART. Emerging real-world evidence also suggests a possible role in carefully selected viraemic individuals with persistent adherence challenges, although such use remains highly targeted.^25^ These advantages are particularly relevant for individuals struggling with daily pill-taking, stigma, or treatment fatigue. However, despite consistent evidence of its efficacy and safety in clinical trials, the real-world feasibility and acceptability of LAI-ART in low-resource settings such as Pakistan remain largely unexplored.

In 2025, the World Health Organization (WHO) recommended LA-CAB/RPV primarily as an alternative switch option for virally suppressed individuals.^26^ More recent International Antiviral Society-United States of America (IAS-USA) has also considered carefully selected viraemic individuals who cannot maintain oral ART despite intensive support, although this remains a highly selective, closely supervised approach.^27^ This global endorsement represents a pivotal step toward expanding equitable access to LAI-ART in resource-limited contexts. It also underscores the urgency of generating context-specific evidence to inform national preparedness, programmatic planning and health system readiness for its potential adoption in Pakistan. While many stakeholders view LAI-ART favourably and believe the strategic investment and policy support could help overcome implementation barriers,^15,28^ some policymakers remain cautious, questioning its added value in settings where oral ART outcomes remain satisfactory.^29^ Despite current eligibility restrictions, evaluating stakeholder perspectives at a preintroduction stage remains important for health system readiness, provider acceptability, infrastructure, and policy alignment. Early exploration of these factors allows for anticipatory planning, identification of implementation barriers, and alignment of new interventions with evolving service delivery models. Therefore, such assessments are relevant even in contexts where the currently eligible population is limited.

Perspectives of PLHIV in Pakistan have also been explored in separate qualitative work, which highlighted both interest in LAI-ART and concerns related to access, adherence to injection schedules, and service delivery requirements.^30^ However, understanding community acceptability alone is insufficient for policy reasons, as successful introduction of LAI-ART also depends on health system capacity, regulatory preparedness, and programmatic feasibility.^15^ Therefore, it is essential to examine the views of healthcare providers and program stakeholders who are directly involved in implementation.

Given Pakistan's unique health system constraints and suboptimal treatment outcomes, assessing healthcare providers’ and program stakeholders’ perspectives is critical to determine the feasibility and acceptability of introducing LAI-ART within the country's HIV care framework. Accordingly, this study explores the perceived feasibility and acceptability of LAI-ART among HIV care providers and programmatic stakeholders in Pakistan.

## Methods

### Study Design and Setting

This qualitative study employed semistructured in-depth interviews with HIV care providers and regulatory representatives. We collected data across major HIV service delivery sites in Islamabad, Rawalpindi, and Karachi, including 3 ART centers at Pakistan Institute of Medical Sciences (PIMS) which is Pakistan's largest HIV referral facility,^31^ Federal Polyclinic’s Maternity Care Hospital , Islamabad and Benazir Bhutto Hospital, Rawalpindi.^32^ Additional participants were recruited from the Infectious Disease (ID) department at PIMS, Agha Khan University Hospital and a local community-based organization (CBO) engaged in HIV service provision. We recruited regulatory representatives from the National AIDS Control program (NACP), the national body overseeing HIV/AIDS control activities.^32^ The Consolidated Criteria for Reporting Qualitative Research checklist is provided in Supplemental Material S1 (S1 File).

### Sampling and Recruitment

We used a purposive sampling strategy to recruit participants representing diverse professional roles and varying levels of engagement in HIV care. This approach was selected to ensure the inclusion of individuals with specialized knowledge and direct experience relevant to HIV treatment, enabling rich, context-specific insights. Following receipt of approval from NACP, recruitment was initiated through both in-person visits to ART centers, the CBO and NACP, as well as online communication with eligible stakeholders. The primary researcher, MWS, a Master of Philosophy (MPhil) Scholar introduced the study, explained its objectives, and emphasized the potential importance of the findings for informing future implementation of LAI-ART in Pakistan. Key informants responded positively and expressed strong interest in participating, noting that such evidence would be essential for preparing the national HIV program for new treatment modalities. Eligible participants included HIV physicians, HIV counsellors, HIV treatment specialists, HIV pharmacists, ID physicians and nursing staff from ART centers, ID department, the CBO, as well as regulatory personnel from NACP. Recruitment continued until data saturation was reached, after which interviews were stopped.^33^

### Interview Guide

The primary researcher (MWS) developed a semistructured interview guide collaboratively with two coauthors (AA and FR). The interviewer MWS had prior familiarity with LAI-ART through literature review, clinical trial reports, and academic teaching sessions, which informed the development of context-relevant questions. Its content was informed by relevant literature assessing feasibility and acceptability of LAI-ART^9,11,12,17^ and was adapted to the Pakistani healthcare context. The guide was refined through expert feedback from all the coauthors and was validated through argumentative and cumulative methods^34^ to ensure clarity, relevance and alignment with study objectives. It included open-ended questions exploring awareness and perceptions of LAI-ART, perceived effects of LAI-ART on adherence, convenience, missed dosing, comparison with oral ART, and suitability for different PLHIV groups, anticipated PLHIV acceptability and related barriers, operational feasibility, resource requirements and potential impacts on stigma and discrimination, as well as recommendations for policy and implementation strategies in Pakistani context. The complete guide is provided in Supplemental Material S2 (S2 File). Particular emphasis was placed on exploring adherence-related issues, including whether LAI-ART could improve adherence compared with daily oral therapy, whether long-dosing intervals could increase missed doses, and which PLHIV groups might benefit most from injectable regimens.

### Data Collection

The lead interviewer (MWS) conducted all interviews from November 2024 to May 2025, applying qualitative research training from his MPhil studies. Conducting interviews by one trained researcher ensured consistency in questioning style and interaction across participants. Prior to data collection, the interviewer had developed substantial familiarity with LAI-ART through extensive literature review, review of clinical trial evidence, and regular academic teaching sessions on LAI-ART as part of postgraduate training activities. This preparation enabled the interviewer to conduct informed and contextually relevant discussions with participants. No prior professional relationship existed between the interviewer and participants, and all participants were approached through professional contacts and permissions after institutional approval. After obtaining informed consent, participants were interviewed at their suggested date and time, and all the interviews were conducted in Urdu language and recorded with a suitable audio recording device. We conducted one online interview through recorded Google Meet session (Google LLC, Menlo Park, California, United States). All the recorded interviews were stored in a restricted-access drive accessible only to the research team, for the purpose of transcription and qualitative data analysis. Interviews were stopped after reaching response saturation.

### Data Analysis

MWS primarily transcribed and translated all the interviews with assistance from MAA, a pharmacy graduate with formal training in qualitative research methods. MWS carefully reviewed the audio recordings and transcripts to ensure accuracy and completeness. Any identifiable information was removed. Any inconsistencies were addressed by cross-referencing the transcripts with the original recordings. Participants did not have access to the transcripts or study findings. Given the exploratory nature of the study, a predefined coding framework was not used, instead, qualitative analysis followed Braun and Clarke's reflexive thematic analysis approach.^35^ Transcripts were deidentified and all participants were assigned unique codes (eg, H1, H2) and compiled into a single document and prepared for manual coding. MWS developed the initial codebook using comment annotations in Microsoft Word. To minimize individual coder bias, transcripts and codes were subsequently reviewed, refined and validated through regular peer debriefing sessions with four research team members AA, MUR, FA, and FR after which the finalized structure was organized into Excel file. Themes and subthemes were generated inductively and subsequently reviewed and verified by all coauthors. Emerging themes were discussed and refined through iterative communications via WhatsApp meetings and emails to ensure consistency and rigor. Illustrative quotations and supporting principal themes are discussed in the results section, with additional excerpts provided in the Supplemental Material S3 (S3 File).

### Ethics Statement

Ethical approval for this study was provided by Riphah Institute of Pharmaceutical Sciences, Riphah International University, Islamabad, Pakistan (Reference No. REC-RIPS/RARE/2024/15). Administrative approval to conduct the study was granted by NACP, Islamabad, Pakistan on the basis of this ethical clearance. An informed consent was obtained from the participants prior to the initiation of the study. All the study procedures adhered to the principles of the Declaration of Helsinki, Good Clinical Practices, and within the applicable laws and regulations of human research in Pakistan.

## Results

Seventeen key informants participated in the study, with a median of 5 years’ experience: 4 ART physicians, 3 ID physicians, 2 ID fellows, 2 NACP HIV treatment specialists, 2 NACP HIV pharmacists, 1 ID nurse and 3 HIV counsellors. Interviews lasted 30 to 90 minutes. Table 1 summarizes the participant characteristics.

**Table 1.** Characteristics of Participants Included in Our Study (*n* = 17).

Participant Number	Gender	Education	Affiliations	Years of Experience	Designation
H1	Female	BSN	ART center, PIMS	19 years	HIV counsellor
H2	Female	FCPS ID	ART center, PIMS	3 years	ART physician
H3	Female	MS in psychology	ART center, PIMS	2 years	HIV counsellor
H4	Female	FCPS ID	ART center, polyclinic	5 years	ART physician
H5	Female	FCPS ID	ID department, PIMS	22 years	ID physician
H6	Female	FCPS ID	ID department, PIMS	2 years	ID fellow
H7	Female	FCPS ID	ID department, PIMS	1 year	ID fellow
H8	Male	MPH	NACP	31 years	HIV treatment specialist
H9	Male	BSN	CBO	2 years	HIV counsellor
H10	Male	MBBS	CBO	1 year	ART physician
H11	Male	MPH	NACP	20 years	HIV treatment specialist
H12	Female	Post-RN	ID ward, PIMS	16 years	ID nurse
H13	Male	MPhil	NACP	15 years	HIV pharmacist
H14	Female	MPhil	NACP	2 years	HIV pharmacist
H15	Male	MS in healthcare management	BBH	4 years	ART physician
H16	Female	FCPS ID	BBH	20 years	ID physician
H17	Male	DABIM (ID)	ID department, AKUH	25 years	ID physician

Abbreviations: ART, antiretroviral therapy; AKUH, Agha Khan University Hospital; BBH, Benazir Bhutto Hospital; BSN, bachelor of science in nursing; DABIM, Diplomate of American Board of Internal Medicine; CBO, community-based organization; FCPS, fellow of college of physicians and surgeons of Pakistan; HIV, human immunodeficiency virus; ID, infectious diseases; MPH: master's in public health; MPhil, master of philosophy; MS, master of science; NACP, National AIDS Control Program; PIMS, Pakistan Institute of Medical Sciences; Post-RN: postregistered nurse.

### Awareness and Understanding of LAI-ART

Participants consistently demonstrated an informed understanding of LAI-ART, recognizing its potential role in HIV care despite its nonavailability in Pakistan's ART centers. They were aware that long-acting treatment involves less frequent dosing than daily regimens.

In Pakistan they are not available at ART centres, here only pills are being given but in Western countries like in Europe and U.S. etc it is being given to improve adherence among those who cannot take pills. (H10: ART Physician)

Participants’ knowledge of specific dosing intervals varied from monthly to yearly options. Informants described learning about LAI-ART through seminars, online resources, professional peer exchanges and discussions with PLHIV (S3 File).

### Perceived Benefits and Opportunities With LAI-ART

#### Community-Level Drivers of LAI-ART Acceptability

Participants perceived strong PLHIV interest and optimism towards LAI-ART noting their awareness of global developments and eagerness for similar options in Pakistan.

For a quite some time people are waiting for this, because it is not us who listen to the news; everyone does. So, they know that something is happening in the world [regarding their treatment], and it is not happening here. They will be very happy [if the injectable is introduced]. (H1: HIV Counsellor)

A few participants considered LAI-ART particularly suitable for PLHIV who inject drugs, citing challenges such as psychological comorbidities and unstable adherence patterns.

For drug users, I believe this is the best option. Because they often have other issues going on simultaneously psychologically and otherwise. (H13: HIV Pharmacist)

Others noted a common perception that injectable therapies are more potent, which could further enhance PLHIV acceptability.

People here have myth that injections are more effective. They request a drip or injection, thinking it will make them get better. (H5: ID Physician)

#### LAI-ART as a Strategic Advancement in the National HIV Response

Given the strategic importance of treatment adherence in HIV care, participants consistently described LAI-ART as a promising additional option. They perceived LAI-ART not merely as an alternative formulation, but as a possible supportive strategy to one of the most persistent challenges in HIV programs, ie, nonadherence.

I think that the whole epidemic response will be turned around. The major issue in PLHIV is loss to follow up, through this we will overcome this gap. (H11: HIV Treatment Specialist)

In addition, participants linked LAI-ART to broader public health targets. An HIV pharmacist noted that the availability of injectables could facilitate more effective implementation of 95-95-95 global HIV targets.

If long-acting injectables become available, then the 95-95-95 global HIV target can be implemented more effectively. (H13: HIV Pharmacist)

While acknowledging that available regimens are generally effective, one HIV care provider highlighted the lack of therapeutic diversity and emphasized the need for additional options. In this context, LAI-ART was viewed as an important addition rather than replacement, strengthening existing treatment framework.

I think it's a good option. The thing is, we as already we don't have a lot of options in terms of treatment for HIV in Pakistan, we have limited availability of drugs. Most of them that we have do work well, but this would give us one more addition, a measure. (H17: ID Physician)

#### Increased Treatment Satisfaction

Participants viewed LAI-ART as a relief from burden of daily therapy thus improving overall comfort.

It's a good option because HIV is a lifelong disease and I do see a lot of patients with pill fatigue who would want to get, you know, other alternatives as opposed to daily pills. (H17: ID Physician)

They believed less dosing could reduce gastrointestinal and other AE associated with oral therapies.

Oral pills cause nausea, vomiting and diarrhoea, so many of these issues would be eased with the injectable. (H11: HIV Treatment Specialist)

#### Increased Confidentiality

Participants viewed LAI-ART as potentially reducing stigma by eliminating visible pill bottles and decreasing risk of inadvertent disclosure.

It will reduce stigma. People often hide their pill bottles, putting them in wrappers or multivitamin containers, so an injectable would lessen this. (H5: ID Physician)

#### Reduced Provider Workload

A subset of participants anticipated operational benefits, suggesting that LAI-ART may reduce administrative workload and allow staff to focus more on adherence support and follow-up monitoring.

This means the workload on HR will decrease. They will have more time to review the surveillance system, identify clients due for follow-up and remind them when their medicines are due. (H8: HIV Treatment Specialist)

### Perceived Barriers to LAI-ART Implementation

#### Travel and Logistical Burden for PLHIV

Participants noted that frequent clinic visits for injections may be impractical for PLHIV from remote areas or those working abroad who depend on multimonth dispensing.

It will be challenging for people from remote areas. Many patients working in Dubai collect six months to a year of pills and do not return during that time. For them, pills are more convenient, coming every month for an injection would be difficult. (H5: ID Physician)

Others suggested that longer-interval formulations (eg, 6 monthly or yearly) would be more feasible (S3 File).

#### Needle Phobia and Injection Reactions

Some participants anticipated resistance to the injectable due to needle phobia and perceived pain.

Needle and pain will be the factor [for convincing the PLHIV]. Otherwise, it is very convenient. (H10: ART Physician)

A few mentioned the potential for severe anaphylaxis, which could pose significant safety concerns to PLHIV.

But there is a chance for anaphylactic reaction. With tablets it hasn’t occurred but there is a chance with injectables. (H16: ID Physician)

#### Stigma and Confidentiality Concerns

A minority of participants noted that clinic-based injections could compromise confidentiality and increase stigma compared to oral medications.

A patient who doesn’t want society to know about his HIV status, he will have to go to a technical person to get the injection, which could affect his confidentiality. (H14: HIV Pharmacist)

#### Forgetfulness and Missed Appointments

Participants noted that with LAI-ART adherence could still be an issue if PLHIV forget their scheduled doses leading to treatment interruptions and drug resistance.

Injectable therapy is offered once patient is virally suppressed with oral pills. But if he stops the treatment and starts it again, there are chances of resistance. (H6: ID Fellow)

Others emphasized that such regimens may be unsuitable for individuals with poor adherence, though feasible for motivated and responsible PLHIV.

You know I would not give this to somebody who is already non adherent to medicines or the treatment. (H17: ID Physician)

Taken together, these responses suggest that participants viewed LAI-ART as more suitable for individuals who are already stable on treatment and able to maintain regular follow-up, rather than those with ongoing adherence difficulties. This interpretation reflects participants’ emphasis on viral suppression prior to initiation, the risk of missed injections, and the need for responsible and motivated PLHIV.

### System and Infrastructure Readiness

#### Training and Staffing Needs

Participants highlighted the need for staff training and additional personnel to safely administer and manage injectable therapy.

Major requirement in training and staffing is expected. The whole counselling process will be changed. In all ART centres, at least two people will be trained, the data entry operator and the counsellor. (H11: Treatment Specialist)

Gender considerations were also highlighted, as the absence of female providers could pose challenges for administering gluteal injection to female clients.

There could be a gender issue also, for example, if the injecting person is a male and has to inject a female patient and vice versa, so this may be an issue. (H17: ID Physician)

One of them noted that most ART staff lack injection experience, raising concerns about handling reactions and new technologies like auto-disable syringes:

Is our staff equipped with training in the use of the auto-disable syringe? Because most capable healthcare providers also require full-fledged training on the use of auto-disable syringe. So, how much our typical ART centre staff will be aligned with this new technology? (H8: Treatment Specialist)

#### Storage and Cold Chain Requirements

Participants anticipated major logistical adjustment for LAI-ART, including cold chain maintenance, secure transportation and potential warehouse expansion to prevent wastage:

There will be major changes, such as expanding the warehouse. Injectable vials can break, and due to handling issues from shipment to facility, 10%–15% may be wasted. Temperature requirements will also need to be maintained. (H11: HIV Treatment Specialist)

#### Facility Infrastructure, Space, and Emergency Readiness

Clinical and regulatory workers highlighted the need for dedicated private spaces to ensure safe and confidential administration for LAI-ART:

Also, we would need a special room or designated space for administering the injection, particularly if it needs to be given in the gluteal muscle. It should be a private area to ensure proper access to the injection site and maintain the patient's dignity and comfort. (H15: ART Physician)

In addition, participants underscored the importance of equipping these areas with emergency setups to manage potential adverse reactions like anaphylaxis:

We have to ensure presence of emergency tray all over the centres, which include airway, endotracheal tube, intubation facility, adrenaline, steroidal injections, trained doctor and nurse who could properly handle the emergency situation with the test/first dose. (H11: Treatment Specialist)

#### Waste Management

Biomedical waste management emerged as a critical concern. A few participants emphasized the need for stricter infection prevention and disposal systems to prevent accidental exposure:

…our major issue is waste management. If an HIV patient is injected and the syringe is not discarded properly, it will be a time bomb that is being thrown in the market. So, ensuring that injections are properly channelled into waste management systems and safely incinerated will be a big challenge that would require action. (H8: Treatment Specialist)

### System Sustainability and Sociocultural Preparedness

#### Funding and Cost Implications

Financial sustainability was the most frequently cited systems-level concern. Participants noted that LAI-ART could substantially increase program costs:

If you're offering this service free of cost to patients, it could mean that the same budget which currently covers 100% of oral medications might only cover 20% if switched to injectables. So that's definitely a cost disadvantage, and we can consider it a major challenge as well. (H13: HIV Pharmacist)

In contrast, strategies including government ownership, centralized procurement and sustained domestic funding were seen as essential to support long-term feasibility:

Sooner or later, we have to opt for domestic funding. We did it during the COVID, all the vaccines were domestically funded …. The government should be the only buyer; government should own it. Private organizations must not be paid. (H11: Treatment Specialist)

#### Sociocultural and Ethical Consideration

Participants anticipated initial hesitation and myths surrounding injectable therapy, noting that shifting community mindsets would require time and consistent counselling.

In Pakistan, changing community mindsets takes time. Transitioning patients from oral medications to injectables and providing the necessary counselling will require a gradual process. (H4: ART Physician)

Several participants emphasized that preserving PLHIV choice in treatment enhances adherence and acceptance.

If we force injectables on a patient who isn’t interested, it could lead to non-adherence. (H15: ART Physician)

#### Need for Pilot and Phased Rollout

Participants strongly endorsed pilot implementation in select ART centers to evaluate feasibility, infrastructure needs and adherence outcomes followed by a phased national rollout:

All NACP-run centres in Pakistan should conduct a pre-launch assessment to evaluate resources, staffing needs, funding requirements and training. Following implementation, a post launch assessment should be carried out to monitor progress and address gaps. (H2: ART Physician)

A cost-benefit analysis was regarded as essential to inform financial decision-making.

For injectables, I would suggest conducting a cost-benefit analysis first. Only after such an analysis should implementation be considered, especially in a country like Pakistan. (H13: HIV Pharmacist)

#### Digital and Health Information Systems for Monitoring

Participants stressed the role of digital tools including mobile applications and management information systems (MIS), in supporting reminders, tracking missed doses and distributing client loads across facilities:

MIS is very important; it can help us manage the load of patients. More importantly, MIS can tell us where the load is high, and we can direct other patients towards low volume centres. We can link the patient with the MIS so that it can give reminder to him and he doesn’t miss a dose and can get injection from any centre. We will digitalize it…. (H11: Treatment Specialist)

#### Integration into Existing Healthcare Models

Participants discussed integrating LAI-ART into multiple delivery models, including outreach programs, community-based networks and primary care centers:

For this, the best approach is outreaching services that go door to door. They can similarly inject people at their homes; this will save expenses of patients. Especially transgender people, who do not go to the hospital due to issues with privacy. Secondly since these community mostly come here, CBOs can be another option. Third one is they can be connected with BHUs for getting injections. (H10: ART Physician)

Importantly, participants agreed that both oral and injectable treatment options should remain available to ensure choice and inclusivity.

In this regard, both arms [of treatment] should be available; in our country, those who are taking oral should be treated side by side and those who are well educated, well aware and understand that they should do it [take injections], for them a separate arm should be introduced. Means you cannot phase out oral medications in Pakistani context. (H5: ID Physician)

Others emphasized on adaptation of differentiated service delivery (DSD) models and establish clear national protocols before implementation (S3 File).

#### Strengthening Counselling Systems

Participants regarded trained counsellors as central to LAI-ART uptake, citing their existing rapport with PLHIV and their role in explaining treatment transition and adherence requirements.

Because with counsellors a very good trustful relationship of patients is already built-up and would further develop if the counsellor has good knowledge about the product, he will give a detailed knowledge of that. (H3: HIV Counsellor)

#### Provider Optimism and Preparedness

Most participants expressed optimism regarding the introduction of LAI-ART, provided that adequate guidelines, training and facilitation structures are established.

Yes, it will be ready provided that a proper proposal is given to the whole team with proper guidelines and they are facilitated in a systematic way. Then we can easily do it; there won’t be any issue. (H16: ID Physician)

However, one care provider cautioned that current limitations in staffing and infrastructure may hinder immediate readiness (S3 File).

## Discussion

This study offers first systematic exploration of acceptability, perceived opportunity and implementation considerations surrounding LAI-ART among key informants in Pakistan, where such regimens are not available. Participants recognized LAI-ART's potential to address longstanding challenges in HIV care, including adherence difficulties, pill fatigue, stigma and treatment satisfaction but also identified substantial system-level barriers ranging from logistical, workforce, infrastructural, cost and sociocultural challenges that must be addressed to support future adoption. By capturing perspectives of providers and regulatory stakeholders at a preintroduction stage in a resource limited, high stigma context, this work provides early insights to inform policy planning, DSD adaptations and phased implementation strategies.

A key consideration in interpreting these findings is the current structure of Pakistan's HIV care cascade. With substantial gaps in diagnosis, treatment coverage, and viral suppression,^20,36^ the proportion of PLHIV currently eligible for LAI-ART under existing clinical guidelines remains limited. Participants in this study demonstrated awareness of this constraint, often describing LAI-ART as primarily suitable for stable, virally suppressed individuals, while not excluding its potential role in selected patients with persistent adherence challenges. This highlights an important distinction between perceived future value and present-day applicability within the existing healthcare system. Crucially, a central observation of this study was stakeholder recognition that, under existing resource constraints, premature large-scale investment in LAI-ART could reduce overall treatment coverage if not supported by additional financing. Accordingly, LAI-ART should be viewed as a potential future addition to existing services rather than a replacement for current oral ART programs. A further important observation is the balance between perceived benefits and implementation realities. While participants highlighted advantages such as reduced pill burden, improved confidentiality, and potential adherence support, these were consistently discussed alongside major structural constraints. Concerns related to cost sustainability, infrastructure limitations, workforce capacity, cold-chain requirements, and infection prevention systems suggest that the introduction of LAI-ART in Pakistan would require careful planning and health system strengthening. These findings indicate that enthusiasm for LAI-ART was conditional, with stakeholders emphasizing phased implementation and alignment with existing program capacity rather than immediate large-scale adoption.

Participant optimism about improved adherence, reduced pill burden and stigma, and minimized AEs, are consistent with findings from HICs^9^ and LMICs.^17,37^ However, in the present study they also emphasized that such optimism is shaped by culturally rooted preferences for injectable therapies, underscoring the need for clear communication to prevent misconceptions about potency and expectations of efficacy.

We found several barriers to LAI-ART implementation including cost and accessibility, needle phobia and injection-related AE, stigma and confidentiality concerns and potential adherence lapses, particularly among individuals already struggling with daily ART or those facing long travel distances to clinics. These barriers reflect both contextual and individual-level challenges that may influence LAI-ART uptake. Cost and accessibility are significant concerns, particularly for PLHIV who travel long distances or rely on multimonth oral refills, mirroring challenges reported in Kenya among adolescents and young PLHIV with competing academic commitments.^38^ Needle phobia and injection-related AEs particularly emerged as notable barriers, aligning with evidence from multiple LMICs including Vietnam^17^ and Uganda.^39^ In Vietnam and Uganda, concerns about pain and side effects contributed to hesitancy and discontinuation, though education and counselling were shown to mitigate these barriers.^11,37,39^

Although LAI-ART may reduce pill-related stigma, our key informants cautioned that frequent visits for injections could heighten confidentiality risks in high stigma-settings, an issue also documented in Dominican Republic and Tanzania.^40^ Anticipated stigma may reduce uptake and retention in care, suggesting the need for confidential and flexible service delivery for population already facing social risk. Concerns about lapses in adherence due to infrequent dosing were similarly shared. Echoing findings from Vietnam, some participants argued that individuals who are already nonadherent to daily ART may be at a higher risk of missing injections.^11^ This issue is particularly relevant in LMICs, where health system limitations may amplify the risk of treatment interruptions,^16^ despite modelling evidence suggesting that prioritizing LAI-ART for individuals with poor adherence could be cost-effective.^41^

Participants emphasized that successful implementation in Pakistan would require substantial infrastructural, logistical and financial preparation. These recommendations reflect observations from Vietnam, Uganda and Kenya, where providers highlighted the high cost of consumables, staffing, cold chain maintenance and reminder systems.^17,37,38^ Even in high income settings, cost-related obstacles remain substantial.^9,13^ A few informants highlighted the risk of anaphylaxis with injectable ART, noting that implementation would require additional staff, space and anaphylaxis response kits, similar to observations made by HIV care providers in Vietnam. This concern represents a unique finding in our study. Financial feasibility emerged as one of the most critical barriers, with a participant warning that introducing LAI-ART could significantly reduce treatment coverage under existing budgets. This concern reflects a fundamental trade-off between innovation and equity in resource-constrained settings, where allocating resources to high-cost interventions may limit access to standard oral ART. For Pakistan, the challenge is therefore not only whether LAI-ART is clinically valuable, but whether its adoption can occur without compromising broader population-level treatment. Injectable regimens are expected to surpass the cost of generic oral ART,^42^ reinforcing the need for cost-effective strategies and robust financing mechanisms. Although WHO endorsement of LA-CAB/RPV provides a policy pathway,^26^ regulatory stakeholders emphasized that centralized procurement, sustainable domestic funding and formal cost-benefit analyses are essential before national uptake. Consequently, this finding warrants broader consideration of the financial and operational requirements for equitable implementation.

Participants also stressed the need for a proper waste management system to ensure the safe LAI-ART introduction, noting that poorly handled injectable waste could increase community HIV exposure. This concern reflects broader infection prevention and control challenges in Pakistan, including irregular training, inconsistent guideline implementation, limited monitoring^43^ and past outbreaks in Larkana (2019)^44^ and Taunsa (2025).^45^ Persistent gaps in hazardous healthcare waste disposal and regulatory oversight underscore the importance of reinforcing waste management as a critical component of national HIV care program for successful LAI-ART implementation.

Sociocultural barriers such as misinformation, hesitance and mistrust were discussed, and similar patterns have been documented in other LMICs.^11,12,38^ Participants’ support for PLHIV choice between oral and injectable options aligned with findings from Vietnam and Kenya.^11,38^ Many recommended phased pilot implementation guided by readiness assessments, consistent with global LMIC recommendations.^11,38,46^ Strengthening service delivery emerged as a key priority, particularly through DSD models which, as highlighted by Grimsrud et al, can be leveraged to extend LAI-ART to populations with ongoing adherence challenges. DSD focused adaptations, such as decentralizing injections to community or lay providers, integrating mobile/outreach clinics, and expanding private pharmacy networks, present practical pathways for expanding access with resource-constrained settings.^47^ These considerations are consistent with broader implementation science perspectives. Using the Practical, Robust Implementation and Sustainability Model Kanazawa et al. emphasized that successful LAI-ART introduction depends not only on clinical efficacy, but also on recipient characteristics, organizational readiness, external environment, and infrastructure. Key unanswered questions include acceptability among diverse and underserved populations, real-world costs to PLHIV, provider preparedness, and ethical concerns such as coercive use among vulnerable groups.^29^ These issues are highly relevant to Pakistan, where stigma, geographic inequity, and constrained health system capacity^20^ may strongly influence uptake, retention, and sustainability of LAI-ART services.

Rather than positioning LAI-ART as a solution to foundational gaps in the HIV care cascade, our findings suggest that its potential role in Pakistan would be incremental and complementary. Consistent with global implementation literature conceptualizing LAI-ART within DSD frameworks,^47^ participants emphasized maintaining standard oral ART while cautiously considering injectable therapy as an additional option. Given current WHO recommendations restricting LAI-ART to virally suppressed individuals,^26^ its relevance in Pakistan may be limited; however, emerging real-world evidence suggests a potential role in carefully selected viraemic individuals with persistent adherence challenges. A compassionate use series reported that 57% of viraemic PLHIV achieved viral suppression following LA-CAB/RPV initiation,^48^ while implementation data from Ward 86 demonstrated sustained suppression in a majority of highly vulnerable patients despite baseline viraemia and social instability.^49–51^ These findings have informed recent updates in international guidance, including IAS-USA,^27^ and the U.S. Department of Health and Human Services,^52^ which now allow consideration of LAI-ART in select viraemic individuals under strict clinical criteria. Careful eligibility screening should also include hepatitis B virus (HBV) status, as currently available long-acting regimens do not provide HBV-active therapy.^53^ Individuals who are unvaccinated should be offered HBV vaccination, while those with chronic HBV infection require careful assessment of ongoing HBV treatment needs before any switch from HBV-active oral ART.^54^ In this context, early stakeholder engagement especially in Pakistan remains important, where retention in care and viral suppression rates remain suboptimal, as it allows phased preimplementation planning and helps align potential introduction of LAI-ART with health system readiness and evolving viral suppression rates. However, this expanded use requires caution. The prolonged pharmacokinetic tail of LA CAB/RPV may result in subtherapeutic drug exposure if injections are delayed or discontinued, increasing the risk of resistance, particularly affecting both nonnucleoside reverse transcriptase inhibitor and integrase strand transfer inhibitor classes.^25^

Pakistan's experience with public–private partnerships (PPPs) demonstrates high treatment success, exemplified by the national tuberculosis program, where nongovernmental organization and general practitioner facilities reported 94% to 96% of success with an overall 90.6% success among PPP-notified cases.^55^ In HIV care, PPPs enhance outcomes by improving logistics, supply-chains and costs efficiency, thereby expanding access in underserved areas.^56^ Broadly PPPs can strengthen health systems by increasing resources, building local expertise, institutionalizing interventions, expanding laboratory and service capacity, while enhancing national ability to mobilize funds for priority programs.^57^ These insights suggest that PPP models could support LAI-ART delivery within Pakistan's mixed health system.

Participants also emphasized on system sustainability through digital tools (eg, mobile apps, MIS) to support adherence monitoring, appointment reminders and service efficiency, consistent with global evidence on digital health interventions.^15^ Consistent counselling was viewed as critical facilitators for uptake in LMICs,^11,46^ while concerns regarding feasibility in underresourced settings parallel cautionary findings from Senegal and Vietnam.^12,38^

Similar considerations were also observed in a separate qualitative study conducted among PLHIV in Pakistan conducted as a part of the same research project, where participants expressed interest in LAI-ART due to reduced pill burden, improved convenience, and potential benefits for privacy and adherence, but also highlighted concerns related to travel requirements, service accessibility, and the need for consistent counselling and reminder systems.^30^ These findings suggest that acceptability at the community level is closely linked to reliable service delivery, flexible care models, and clear communication, reinforcing the importance of system readiness alongside PLHIV-level support for successful implementation of LAI-ART.

Our study included pharmacists from the national HIV supply chain, a group seldomly represented in qualitative research on LAI-ART in LMICs. Pharmacist contribute meaningfully to HIV care by boosting behavioral, clinical,^58^ economic, and humanistic outcomes.^59^ Evidence from Pakistan demonstrates that even brief pharmacist-led interventions significantly improve adherence and immunological outcomes, including increased CD4 counts and substantial gains in adherence behavior within short follow-up periods. These improvements are mediated through targeted counselling that addresses context-specific barriers such as forgetfulness, stigma, mobility constraints, and misconceptions about ART, while strengthening adherence self-efficacy, and medication beliefs.^60^ Beyond adherence support, pharmacists function as medication therapy managers, with competencies in identifying drug–drug interactions, interpreting resistance patterns, and optimizing ART regimens through collaborative practice models. These capabilities are particularly critical for LAI-ART, where eligibility assessment, resistance screening, and management of delayed or missed injections require advanced pharmacotherapeutic insight.^61^ In this context, pharmacists can play a critical role in reinforcing visit adherence, managing missed-dose protocols, and maintaining longitudinal engagement with PLHIV.

From a health systems and policy perspective the economic evidence is particularly compelling. Pharmacist-led interventions in Pakistan yield higher quality-adjusted life years (QALYs) at an incremental cost-effectiveness ratio of 1383 USD/QALY, remaining below the national willingness-to-pay threshold, demonstrating clear cost-effectiveness.^59^ Broader economic evaluations further demonstrate that pharmacist-managed programs reduce downstream costs by decreasing hospitalizations, opportunistic infections, and HIV transmission, with reported return-on-investment ratios up to 2.96:1 and substantial long-term savings.^62^ When extrapolated to LAI-ART, these economics gains are likely to be amplified. Although LAI-ART involves higher upfront costs related to drug procurement, cold-chain logistics, and administration, pharmacist involvement can mitigate these costs by optimizing PLHIV selection, preventing inappropriate initiation, reducing missed injections, and minimizing costly treatment failures. In resource-limited settings such as Pakistan, where financial and structural barriers are prominent, this shift from reactive to preventive pharmacist-led care models is not optional but necessary.

Operationally, pharmacists can function as coordinators across LAI-ART care continuum. Evidence from pharmacist-led CAB/RPV programs has demonstrated high treatment retention (84%) through structured referral evaluation, eligibility assessment, and continuous follow-up.^63^ Their role in synchronizing procurement, managing supply chains, ensuring timely drug availability^64^ is particularly relevant in Pakistan's centralized HIV program, where stockouts and inefficient logistics coordination remain critical concerns. Additionally, qualitative evidence suggests that decentralizing LAI-ART delivery to pharmacy settings can expand access, reduce clinic burden, and improve convenience, provided that trust, training, and privacy concerns are addressed.^65^ This aligns with the need for DSD models in Pakistan, especially for geographically dispersed or stigmatized populations. These findings highlight the value of pharmacists in maintaining continuity of care and optimizing treatment transitions. In addition, pharmacists can contribute to supply chain reliability, a key requirement for LAI-ART implementation in Pakistan, where workforce-limited and fragmented HIV services persist.^66^ Policy frameworks should therefore move beyond viewing pharmacists as dispensers and formally integrate them into HIV care pathways, particularly within the NACP. This includes developing structured training programs in HIV pharmacotherapy and LAI-ART administration, establishing collaborative practice agreements to enable pharmacists to conduct eligibility assessments and monitor therapy, and embedding pharmacists within multidisciplinary HIV teams. Furthermore, investment in pharmacy-based or pharmacy-linked LAI-ART delivery can serve as a pragmatic solution to workforce shortages and clinic overcrowding, while maintaining continuity of care. Based on stakeholder insights and the broader evidence base, several actions are recommended to guide the phased, equitable and sustainable introduction of LAI-ART in Pakistan (Table 2).

**Table 2.** Recommendations for Future LAI-ART Introduction in Pakistan.

Domain	Recommendations
Policy and regulatory preparedness	Develop national guidelines/SOPs in consultation with all stakeholders comprised of community, HCP and policy makers. Ensure centralized procurement of LAI-ART formulations. Conduct thorough economic evaluations. Plan for sustainable long-term domestic funding to support introduction of LAI-ART in Pakistan Define clear eligibility criteria aligned with current WHO recommendations, evolving international guidance, and national program capacity. Include criteria for careful patient selection, resistance assessment, follow-up capacity, and adherence support prior to enrolment.
Pilot and phased rollout	Begin with targeted pilot studies in select ART centers to gauge preparedness, refine protocols and understand contextual obstacles before a nationwide rollout. Phased introduction should initially prioritize PLHIV under eligible current clinical guidelines for LAI-ART. Any expansion to carefully selected individuals with persistent viraemia and major adherence challenges should occur only in closely monitored pilot settings under stringent criteria
Health system infrastructure	Equip healthcare workers with skills in injection administration, managing AE and counselling for injectable regimens, while ensuring staffing reflects gender-appropriate considerations.
Digital and information systems	Strengthen MIS and mobile-based reminder systems to track appointments, send missed-dose alerts and manage PLHIV flow, including reminders for scheduled injection visits, supporting more effective ART delivery in the future.
Community engagement and counselling	Develop culturally sensitive educational strategies to address myths, stigma and fear while empowering counsellors to guide PLHIV in making informed choices about LAI-ART, with clear counselling on benefits, risks, and follow-up requirements.
Adherence and safety systems	Establish protocols to monitor adherence, manage missed doses and ensure injection safety before rolling out LAI-ART, including protocol-based management of missed injection visits. Ensure oral bridging or alternative ART pathways when injections are delayed, interrupted, or discontinued.
Safe IPC protocols	Establish and enforce rigorous IPC protocols, including safe injection practices, sharps disposal and management of injection-related risks, to ensure PLHIV and healthcare worker safety during LAI-ART delivery
Public–private partnerships for LAI-ART delivery	Foster collaboration between public and private healthcare sectors to expand access, strengthen supply chains, extend service coverage to underserved areas, build local capacity and support sustainable delivery of LAI-ART in Pakistan.
Integrating pharmacists into LAI-ART services	Leverage pharmacists as key members of the HIV care teams to enhance PLHIV counselling, adherence management, monitoring for AE and safe injection practices. Engage pharmacists in procurement oversight, stock management, cold-chain monitoring, and treatment continuity planning.
Future research needs	Conduct readiness assessments, evaluate user acceptability and carry out implementation research specifically tailored to the Pakistani context. Evaluate outcomes, missed-dose patterns, resistance emergence, and cost-effectiveness in early implementation phases. Evaluate LAI-ART implementation across diverse PLHIV subgroups, including acceptability, uptake, retention, provider readiness, people-reported outcomes, and ethical considerations.

Abbreviations: AE, adverse effects; ART, antiretroviral therapy; LAI-ART, long-acting injectable antiretroviral therapy; MIS, management information system; IPC, infection prevention and control; PLHIV, people living with HIV; HCP, healthcare provider.

### Strengths and Limitations

This study offers multilevel insights by incorporating perspectives of both HIV care providers and regulatory stakeholders providing a comprehensive view of factors influencing future LAI-ART implementation in Pakistan. Inclusion of diverse cadres in HIV care including pharmacists strengthened the contextual relevance of the findings for LMIC health systems.

Limitations include the focus on selected urban settings, which may not capture the perspectives of providers in rural or more resource-limited areas. Moreover, participants’ views were based on hypothetical scenarios rather than direct experience with LAI-ART, which may limit the accuracy of perceived feasibility and acceptability. Importantly, this manuscript reflects provider and regulatory stakeholder perspectives only and did not include direct interviews with PLHIV; therefore people-level preferences, concerns, and lived experiences are not directly represented in the present analysis. However, this work forms part of a broader research project in which PLHIV perspectives on LAI-ART acceptability in Pakistan were explored in a separate qualitative study, providing complementary people-level insights.^30^ Future research including pilot implementation, economic assessments, subgroup acceptability studies, ethical evaluations, and longitudinal evaluations with both providers and PLHIV is essential to generate real-world evidence and inform sustainable national adoption of LAI-ART. Overall, these findings should be interpreted as exploratory and preimplementation in nature, and do not indicate immediate programmatic readiness for widespread LAI-ART rollout in Pakistan. Rather, they highlight priorities for phased planning, further evaluation, and health systems strengthening.

## Conclusions

This study provides an early and contextually grounded assessment of the feasibility and acceptability of future LAI-ART rollout in Pakistan, capturing the perspectives of HIV care providers and regulatory representatives. Our participants view LAI-ART as a promising future option to improve adherence, reduce pill burden and enhance confidentiality for PLHIV, but also identified substantial barriers that must be addressed before implementation, including high costs, infrastructural limitations, cold-chain and storage requirements, staff training needs and sociocultural hesitations. Participants noted that frequent clinic visits for injections may disadvantage PLHIV who rely on multimonth refills or live far from ART centers.

If introduced in Pakistan, the initial role of LAI-ART would likely be limited to carefully selected individuals, primarily those who are virally suppressed, with potential consideration for a small subgroup with persistent viraemia related to major adherence challenges under strict clinical criteria and close monitoring, and should be considered a complementary option within the existing framework rather than a replacement for oral ART. Stakeholders emphasized that successful introduction would require phased implementation, economic evaluation, strengthened counselling systems, and improved service delivery capacity. Integration through DSD models, digital adherence support, and community-based approaches, while ensuring PLHIV choice and alignment with existing program capacity, will be important for sustainable and equitable implementation of LAI-ART in Pakistan. These exploratory findings primarily inform early planning priorities rather than immediate large-scale implementation.

## Supplemental Material

sj-docx-1-jia-10.1177_23259582261452317 - Supplemental material for Feasibility and Implementation Considerations for Long-Acting Antiretroviral Therapy in Pakistan: A Qualitative Study of Healthcare Providers and Programmatic Stakeholders Supplemental material, sj-docx-1-jia-10.1177_23259582261452317 for Feasibility and Implementation Considerations for Long-Acting Antiretroviral Therapy in Pakistan: A Qualitative Study of Healthcare Providers and Programmatic Stakeholders by Muhammad Wasay Shahid, Masood-ur-Rehman , Faizur Rehman, Mehran Riaz, Muhammad Aaqib Akram, Farah Azhar, Malik Muhammad Umair, Nasim Akhtar, Musarat Jabeen and Ali Ahmed in Journal of the International Association of Providers of AIDS Care (JIAPAC)

sj-docx-2-jia-10.1177_23259582261452317 - Supplemental material for Feasibility and Implementation Considerations for Long-Acting Antiretroviral Therapy in Pakistan: A Qualitative Study of Healthcare Providers and Programmatic Stakeholders Supplemental material, sj-docx-2-jia-10.1177_23259582261452317 for Feasibility and Implementation Considerations for Long-Acting Antiretroviral Therapy in Pakistan: A Qualitative Study of Healthcare Providers and Programmatic Stakeholders by Muhammad Wasay Shahid, Masood-ur-Rehman , Faizur Rehman, Mehran Riaz, Muhammad Aaqib Akram, Farah Azhar, Malik Muhammad Umair, Nasim Akhtar, Musarat Jabeen and Ali Ahmed in Journal of the International Association of Providers of AIDS Care (JIAPAC)

sj-docx-3-jia-10.1177_23259582261452317 - Supplemental material for Feasibility and Implementation Considerations for Long-Acting Antiretroviral Therapy in Pakistan: A Qualitative Study of Healthcare Providers and Programmatic Stakeholders Supplemental material, sj-docx-3-jia-10.1177_23259582261452317 for Feasibility and Implementation Considerations for Long-Acting Antiretroviral Therapy in Pakistan: A Qualitative Study of Healthcare Providers and Programmatic Stakeholders by Muhammad Wasay Shahid, Masood-ur-Rehman , Faizur Rehman, Mehran Riaz, Muhammad Aaqib Akram, Farah Azhar, Malik Muhammad Umair, Nasim Akhtar, Musarat Jabeen and Ali Ahmed in Journal of the International Association of Providers of AIDS Care (JIAPAC)

sj-docx-4-jia-10.1177_23259582261452317 - Supplemental material for Feasibility and Implementation Considerations for Long-Acting Antiretroviral Therapy in Pakistan: A Qualitative Study of Healthcare Providers and Programmatic Stakeholders Supplemental material, sj-docx-4-jia-10.1177_23259582261452317 for Feasibility and Implementation Considerations for Long-Acting Antiretroviral Therapy in Pakistan: A Qualitative Study of Healthcare Providers and Programmatic Stakeholders by Muhammad Wasay Shahid, Masood-ur-Rehman , Faizur Rehman, Mehran Riaz, Muhammad Aaqib Akram, Farah Azhar, Malik Muhammad Umair, Nasim Akhtar, Musarat Jabeen and Ali Ahmed in Journal of the International Association of Providers of AIDS Care (JIAPAC)
