# Supplementary material for: Feasibility and Implementation Considerations for Long-Acting Antiretroviral Therapy in Pakistan: A Qualitative Study of Healthcare Providers and Programmatic Stakeholders
Source: J Int Assoc Provid AIDS Care. 2026 May 19;25:23259582261452317. doi: 10.1177/23259582261452317 (PMC13527321; doi:10.1177/23259582261452317)
Supplement: Supplementary file 1 [file sj-docx-1-jia-10.1177_23259582261452317.docx]

**Supplemental Material**

**Supplemental Material 1 (S1 File). Consolidated Criteria for Reporting Qualitative Research (COREQ) Checklist**

**Description:** This file contains the completed COREQ 32-item checklist. Page numbers and relevant excerpts of the manuscript indicate where each reporting item is addressed in the manuscript.

**Supplemental Material 2 (S2 File). Semi-Structured Interview Guide for Care Providers and Regulatory Stakeholders in HIV Care**

**Description:** This file contains Semi-structured interview guide that was used to conduct interviews from HIV care providers and regulatory Stakeholders in HIV care.

**Supplemental Material 3 (S3 File). Themes, Subthemes and Relevant Snippets**

**Description:** This section presents the relevant snippets under the overarching themes and subthemes generated after the thematic analysis
