# Supplementary material for: Feasibility and Implementation Considerations for Long-Acting Antiretroviral Therapy in Pakistan: A Qualitative Study of Healthcare Providers and Programmatic Stakeholders
Source: J Int Assoc Provid AIDS Care. 2026 May 19;25:23259582261452317. doi: 10.1177/23259582261452317 (PMC13527321; doi:10.1177/23259582261452317)
Supplement: Supplementary file 2 [file sj-docx-2-jia-10.1177_23259582261452317.docx]

**Supplemental Material 1 (S1): Consolidated Criteria for Reporting Qualitative Research (COREQ) Checklist**

| **Item No** | | **Guide Questions/Description** | **Explanation** | **Reported on Page #** | **Relevant text from manuscript/Section Name** |  |  |  |
| --- | --- | --- | --- | --- | --- | --- | --- | --- |
| **Domain 1: Research team and reflexivity** | | | | | |  |  |  |
| **Personal Characteristics** | | | |  |  |  |  |  |
| 1. Interviewer/ facilitator | | Which author/s conducted the interview or focus group? | Muhammad Wasay Shahid | - | - |  |  |  |
| 2. Credentials | | What were the researcher’s credentials? E.g., PhD, MD | The researcher is an MPhil Scholar in Pharmacy Practice | - | - |  |  |  |
| 3. Occupation | | What was their occupation at the time of the study? | MPhil. Scholar | - | - |  |  |  |
| 4. Gender | | Was the researcher male or female? | The researcher was Male |  |  |  |  |  |
| 5. Experience and training | | What experience or training did the researcher have? | Interviewer Muhammad Wasay Shahid had a full training about qualitative research methods |  |  |  |  |  |
| **Relationship with participants** | | | | | |  |  |  |
| 6. Relationship established | | Was a relationship established prior to study commencement? | Participants were directly approached through professional contacts and references. We explained out objective and nature of our study and after obtaining informed consent we interviewed them | - | - |  |  |  |
| 7. Participant knowledge of the interviewer | | What did the participants know about the researcher? e.g. personal goals, reasons for doing the research? | There was no personal relationship between the participants and the interviewer. It was explained in the informed consent that this study is the part of interviewer’s MPhil project. | - | - |  |  |  |
| 8. Interviewer characteristics | | What characteristics were reported about the interviewer/facilitator? e.g. Bias, assumptions, reasons and interests in the research topic | It was explained that this study is being conducted for research purpose. This research is a part of the interviewer’s MPhil project. No other bias was identified. | - | - |  |  |  |
|  | **Domain 2: study design** | | | | |  |  |  |
|  | **Theoretical framework** | | | | |  |  |  |
|  | 9. Methodological orientation and Theory | What methodological orientation was stated to underpin the study? e.g. grounded theory, discourse analysis, ethnography, phenomenology, content analysis | The researcher used reflexive thematic analysis approach | 6 | Given the exploratory nature… |  |  |  |
|  | **Participant selection** | | | | |  |  |  |
|  | 10. Sampling | How were participants selected? e.g., purposive, convenience, consecutive, snowball | Yes, through purposive sampling | 5 | We used a purposive sampling… |  |  |  |
|  | 11. Method of approach | How were participants approached? e.g., face-to-face, telephone, mail, email | Yes, we approached participants both in-person as well as online communication | 5 | …recruitment was initiated through…. |  |  |  |
|  | 12. Sample size | How many participants were in the study? | 17 care providers and Regulatory Stakeholders | 7 | Seventeen key informants participated… |  |  |  |
|  | 13. Non-participation Setting | How many people refused to participate or dropped out? Reasons? | N/A | - | - |  |  |  |
|  | 14. Setting of data collection | Where was the data collected? e.g., home, clinic, workplace |  | 4 | We collected data across… |  |  |  |
|  | 15. Presence of nonparticipants | Was anyone else present besides the participants and researchers? | No one was present besides the participants and researchers | - | - |  |  |  |
|  | 16. Description of sample | What are the important characteristics of the sample? e.g. demographic data, date | All the important characteristics are discussed in Table 1 | 7-8 | - |  |  |  |
|  | **Data collection** | | | | |  | | **Reported on Page #** |
|  | 17. Interview guide | Were questions, prompts, and guides provided by the authors? Was it pilot tested? | S2 File contains the complete interview guide. The guide was refined through expert feedback from all the co-authors and was validated through argumentative and cumulative methods to ensure clarity, relevance and alignment with study objectives; formal pilot testing was not done. | 5 | The primary researcher (MWS) developed a semi-structured interview guide…. |  |  |  |
|  | 18. Repeat interviews | Were repeat interviews carried out? If yes, how many? | No repeat interviews were carried out | - | - |  |  |  |
|  | 19. Audio/visual recording | Did the research use audio or visual recording to collect the data? | The research used audio recording. | 6 | …and recorded with a suitable audio recording device… |  |  |  |
|  | 20. Field notes | Were field notes made during and/or after the interview or focus group? | No field notes were made during the interview | - | - |  |  |  |
|  | 21. Duration | What was the duration of the interviews or focus group? | Interviews lasted 30-90 minutes. | 7 | Interviews lasted…. |  |  |  |
|  | 22. Data saturation | Was data saturation discussed? | Yes | 5 | Recruitment continued until…. |  |  |  |
|  | 23. Transcripts returned | Were transcripts returned to participants for comment and/or correction? | N/A | - | - |  |  |  |
|  | **Domain 3: analysis and findings** | | | | |  |  |  |
|  | **Data analysis** | | | | |  |  |  |
|  | 24. Number of data coders | How many data coders coded the data? | Muhammad Wasay Shahid coded the entire data, which along with the corresponding excerpts were reviewed, refined and validated by four co-authors. | - | - |  |  |  |
|  | 25. Description of the coding tree | Did the authors provide a description of the coding tree? | Illustrative quotations supporting principal themes and emerging subthemes are discussed in the results section, with additional excerpts provided in the S3 File | - | - |  |  |  |
|  | 26. Derivation of themes | Were themes identified in advance or derived from the data? | Themes were derived from the data | - | - |  |  |  |
|  | 27. Software | What software, if applicable, was used to manage the data? | All the recorded interviews were stored in a restricted-access drive accessible only to the research team. The transcripts were coded using comment annotations in word. All the codes and transcripts were transferred into Excel for analysis. | - | - |  |  |  |
|  | 28. Participant checking | Did participants provide feedback on the findings? | The interviews/findings were not shared with the participants | - | - |  |  |  |
|  | **Reporting** | | | | |  |  |  |
|  | 29. Quotations presented | Were participant quotations presented to illustrate the themes/findings? Was each quotation identified? e.g., participant number | Yes, participant quotations are added to illustrate the findings. We identified each quotation with a participant number and the occupation | 7—14 | Results Section |  |  |  |
|  | 30. Data and findings consistent | Was there consistency between the data presented and the findings? | Yes. There was consistency between the data presented and the findings, as all themes and subthemes were directly derived from the interview data and were supported by representative participant quotations, ensuring that the interpretations were grounded in empirical evidence | 7—14 | Results Section |  |  |  |
|  | 31. Clarity of major themes | Were major themes clearly presented in the findings? | Yes, all the major themes were clearly presented in the findings | - | - |  |  |  |
|  | 32. Clarity of minor themes | Is there a description of diverse cases or a discussion of minor themes? | Yes. While the analysis primarily identified dominant themes, a small number of participants expressed differing views within specific subthemes, and these divergent perspectives were captured either as variations within existing subtheme or as distinct minor subthemes where appropriate | - | - |  |  |  |
