# Supplementary material for: Feasibility and Implementation Considerations for Long-Acting Antiretroviral Therapy in Pakistan: A Qualitative Study of Healthcare Providers and Programmatic Stakeholders
Source: J Int Assoc Provid AIDS Care. 2026 May 19;25:23259582261452317. doi: 10.1177/23259582261452317 (PMC13527321; doi:10.1177/23259582261452317)
Supplement: Supplementary file 3 [file sj-docx-3-jia-10.1177_23259582261452317.docx]

**Supplemental Material 2 (S2 File): Semi-Structured Interview Guide for Care Providers and Regulatory Stakeholders in HIV Care**

**1. Informed Consent**

Hello, my name is Muhammad Wasay Shahid, and I am an MPhil Scholar in Pharmacy Practice from Riphah International University. We are conducting a study to understand your views on acceptability and feasibility of Long-Acting Injectable Antiretroviral Therapy (LAI-ART) in Pakistan HIV care continuum. This research is a part of my MPhil project which will assess acceptability and feasibility of LAI-ART from people living with HIV (PLHIV), HIV care providers and regulatory stakeholders.

Your participation is voluntary. I will ask you some questions about your knowledge of LAI-ART, Perceptions of LAI-ART as treatment option, benefits of LAI-ART among people living with HIV (PLHIV), barriers to LAI-ART access and use, infrastructure requirements and policy recommendations. It is qualitative research which will involve audio interviews. This interview will take some of your time. You can skip any question or stop at any time. With your permission, I would like to record this interview.

Your responses will be kept confidential. Your name will not appear in any report. There are no direct benefits or risks to you, but your input may help improve HIV care.

Do you understand what I have explained?

Do you agree to participate in this interview?

- Yes
- No

May I record this interview?

- Yes
- No

**Signature of the Participant for Physically Available Participants:** _________________

**Participant ID:** ______________

**Date:** ______________________

**For more information regarding this project, please contact the following.**

**Muhammad Wasay Shahid**

**Email:** [muhammadwasayshahid1@gmail.com](mailto:muhammadwasayshahid1@gmail.com)

**Contact:** +923345399124

**2. Demographics**

I. **Interview Settings**
a. Antiretroviral Therapy Centre
b. CBO
c. National AIDS Control Program (NACP)/Provincial AIDS Control Program (PACP)
II. **Type of Participant (Regulatory stakeholder/Care Provider)**
a. Treatment Specialist at NACP/PACP
b. Pharmacist
c. HIV Physician in ART centre
d. Infectious Disease Physician
e. Nurse
f. Counsellor
g. Other (Mention)______________________________
III. **Education:** _________________________________
IV. **Years of Experience:** ________________________

**3. General Awareness**

a. Have you heard about LAI-ART before this interview?
b. Where have you learned about them?
c. What do you know about them?

**4. Perceptions**

a. What are your thoughts on LAI-ART as a treatment option for HIV?
b. How do you think LAI-ART differs from current oral ARVs in terms of effectiveness, convenience and adherence?
c. Is it possible that forgetfulness with next dosing may be increased? Since it is easier to remember daily medicine dosing as compared to LAI-ART.

**5. PLHIV Acceptability and Related Barriers**

a. In your experience how receptive patients would be to LAI-ART (asking more specifically about key populations)
b. What concerns do you anticipate patients might have towards LAI-ART (asking what questions PLHIV would ask)?
c. What would be the patient related barriers that may impact the uptake of this program?
d. Do you think that with this program, PLHIV would engage in more risky behaviours?

**6. Clinical Acceptability**

a. What are the perceived advantages and disadvantages of LAI-ART against daily oral ART for PLHIV?
b. How do you think that introduction of LAI-ART would impact adherence rate among PLHIV?

**7. Operational Feasibility**

a. What changes do you anticipate being made in your healthcare settings to implement LAI-ART?
b. Do you foresee any logistical changes in providing LAI-ART to PLHIV?

**8. Resource Allocation**

a. How do you think that LAI-ART would impact resources in your settings (e.g. storage, training, staff etc.)
b. What would be the basic requirements you think would be needed to support this program at your ART centre/CBOs (funding, budgets, technical support)?
c. What additional support or resources would be necessary for the implementation of LAI-ART in your healthcare settings?
d. Do you think LAI-ART would be more cost-effective for patients? Like patients would have to bear less expenses on travel to other cities, accommodation, food, and leave from work?

**9. Stigma and Discrimination**

a. How would you think LAI-ART would improve or hinder the efforts to reduce stigma around PLHIV?

**10. Final Thoughts and Policy Recommendations**

a. What are your key recommendations in making LAI-ART a success in PLHIV, especially non-adherent populations, within Pakistani context (especially focusing on Injecting Drug Users living with HIV)?
b. After all this discussion, do you think you or your setting is ready/hopeful for this new form of treatment?
c. What further research or pilot programs would you suggest for LAI-ART implementation?
d. What are the main policy considerations when implicating LAI-ART in Nation HIV/AIDS control strategies?
