# Supplementary material for: Feasibility and Implementation Considerations for Long-Acting Antiretroviral Therapy in Pakistan: A Qualitative Study of Healthcare Providers and Programmatic Stakeholders
Source: J Int Assoc Provid AIDS Care. 2026 May 19;25:23259582261452317. doi: 10.1177/23259582261452317 (PMC13527321; doi:10.1177/23259582261452317)
Supplement: Supplementary file 4 [file sj-docx-4-jia-10.1177_23259582261452317.docx]

**Supplemental Material 3 (S3 File): Themes, Subthemes and Relevant Snippets**

| **Themes** | **Subthemes** | **Snippets** |
| --- | --- | --- |
| 1. Awareness and understanding of LAI-ART |  | **H11**: I had attended a webinar and also heard on LinkedIn that Long-Acting Injectables are going to be launched ….  **H16:** We have groups, ID-related groups, which include ID physicians and other microbiologists, both national and international (including Pakistanis). From those groups, I came to know that work is being done on this topic.  **H1:** I have heard recently that it is in research process. I have heard in Canada etc and also heard about in US, in Florida etc, they are getting injections. But I have heard that they are available as six monthly [injectable], people go for six monthly or once a year to get it. Now, I don’t know, means there are different rumours and news also. I didn’t have what we call authentic news. What is authentic [news], tell me.  **H12:** I didn’t read in much detail, but I do remember reading that patients do receive Long Acting injectables. That’s where I got the initial introduction.  **H13:** I’ve been working in healthcare and public health for almost 15 years now, and I’m also associated with international organizations. From time to time, I participate in various meetings, where we keep updating our knowledge. That’s how I came to know about them.  **H13:** Yes, since we are working in the AIDS-related program, we have definitely heard about them and know they are available. However, we haven’t conducted any deep research or analysis on them yet.  **H14:** Just a little bit, because they haven't yet become part of our practice.  **H13:** Just like in contraceptives—there are short-acting medicines that need to be taken daily, and there are also long-acting ones that are taken every three months…. So, the difference between long-acting and short-acting lies in how frequently the medicine needs to be taken.  **H17:** …They are given with the current approval as cabotegravir, which is given every 2 months in combination with rilpivirine. I know there's a cold chain associated with it and has good treatment outcomes.  **H5:** Some are six monthly; some are yearly also. About cabotegravir which we heard about that it will be approved for Pakistan was once monthly. |
| 2. Perceived Benefits and opportunities with LAI-ART | Community-Level Drivers of LAI-ART Acceptability | **H17:** Those folks are generally active members of society, often not very old, not used to taking pills to begin with. And if given the option they may actually opt for getting, IM every 2 months.  **H2:** For IDUs it will not be a problem but for other [PLHIVs] it will be a problem…. They will be overjoyed. |
|  | LAI-ART as a Strategic Advancement in the National HIV Response | **H1:** People will remember it, very effectively they will remember it, when they get rid of the pills, they will remember it.  **H10**: So, if a patient gets an injection for a week or for a month, it will definitely be more effective than the tablet which will significantly improve compliance and reduce the loss to follow up. |
|  | Increased Treatment Satisfaction | **H10:** When daily doses are given, they cause toxicity, while injections are given less frequently.  **H1:** Patients who come, taking pills for ten years, ask me; Madam isn’t there a treatment, so that we can get rid of these pills. |
|  | Increased Confidentiality with LAI-ART | **H8:** Number two, every patient coming each month to get medication is big problem for them, since stigma and all that is attached to it. So, if we [shift] him to twice dose per annum, so patients concern of coming frequently will be curbed.  **H14:** There is a confidentiality factor. A person who previously took medicine daily now has to get an injection once a month. Their confidentiality will be better maintained. |
|  | Reduced Provider Workload with LAI-ART | **H11:** There will be a few busy days and then it will ease off.  **H13:** From the caregivers’ perspective, this method would actually be easier. It simplifies the treatment process for them and helps ensure that the patient remains adherent to treatment more effectively |
| 3. Perceived barriers to LAI-ART Implementation | Travel and Logistical Burden for PLHIV | **H10:** And the biggest of all is availability if it is available far away because Oral [pills] can be easily obtained, so it depends upon this.  **H5:** Coming after six months will be easier, if there is an option for a six-monthly injection, then it is very easier for him.  **H15:** That’s a very important concern. Many of these patients come from remote areas and usually take medication for 6–7 months at a time to avoid frequent travel. Now, if they’re required to come every two months just for an injection, it won’t be effective for them. The travel and cost involved would make it difficult.  **H2:** For locals it will be good, for those who come from far-flung areas how would they be adherent. |
|  | Needle phobia and Injection Reactions | **H11:** Number one is drug reaction. With oral there is very slow reaction, but injection has a fast reaction with the first dose.  **H16:** yeah, the patient would think that he got a swelling from this. It also depends on whether we are going to administer it once a month, so would finding that site be difficult. It all depends upon the degree of local site reaction. So, this can be a disadvantage.  **H4:** For injections, it will be a little difficult for them. And injection or needle phobia and all that you know, being injected, is also considered something painful, it will prick |
|  | Stigma and Confidentiality Concerns | **H5:** But still, he will say that I will not go inside the clinic to get it. Give me, I will administer it myself. Sometimes, when I send them to ART centre for registration, they usually ask if they can send someone else because their relative may see them, but they will have to come for the injection.  **H4:** If they are taking 3 months of medications with us, they will take it daily, even if they have to take it in secrecy, they won’t be exposed…to the public that they are getting the injections. |
|  | Forgetfulness and Missed Appointments | **H2:** Yes, this is right. Those who are taking daily have a reflexive reminder but if a person forgets his monthly injection.  **H5:** Patients also forget their daily ones also. So, they [may forget] their monthly ones. It means they are not seriously taking their condition. Because those who are really serious, they will properly remember that on this day I have to get one [shot].  only one dose, not more than one. There is something written about it in the guidelines. |
| 4. System and Infrastructure Readiness | Training and Staffing Needs | **H15:** Firstly, we don’t have trained staff, which is a basic requirement for this kind of service.  **H1**: Yes, for that we have to involve a nurse or a technician, they will be trained, or a new post is to be created. This is the job of logistic cell; they will do it.  **H11**: absence of female provider for giving gluteal injections to female |
|  | Storage and Cold Chain Requirements | **H13**: If a cold chain is required, it would be a major challenge. In Pakistan, cold chain systems are not available everywhere.  **H2:** Storage will be required. You see for storage, we don’t know if we have to maintain a cold chain. |
|  | Facility Infrastructure, Space and Emergency Readiness | **H16:** a separate room for someone who could inject… A new room like retention room will be required.  **H11:** We need a separate area from the OPD, where they could draw blood and give long-acting ARVs, and should have provision for emergency situations like anaphylaxis or other side effect.  **H16:** a crash trolley for anaphylaxis these would be required. |
|  | Waste Management | **H4:** When you are using syringes, how my staff or I have to decontaminate the syringes or what we have to do, we would be needing too many things. This is the technical issue; can you provide that much that how the syringe is being injected into IVs and how it is disposed of.  **H11:** For this you need to see what kind of IPC practices are followed in Pakistan. How they are disinfecting and injecting the medicine into the patient. Otherwise, this disposable waste has potential for transmission. |
| 5. System Sustainability and Sociocultural Preparedness | Funding and Cost Implications | **H13:** One disadvantage is that it would consume a significant part of the budget. It would be quite costly, and you’d need more resources.  **H8:** some cost issue [exists] but I believe that if we procure it in bulk, it becomes cost effective. So, if government implements it as a policy and we have WHO advisory then we don’t think logistics will be a much problem. |
|  | Sociocultural and ethical consideration | **H15:** I remember something similar happened when a new medicine, ELT, was introduced back in 2018. Later, it came under a different name, DLT, in blue packaging. Even though it was the same formulation, patients reported side effects with the new packaging. It was all about their beliefs and trust—not the medicine itself.  **H16:** The authority should lie with the patient to decide what suits them. Because in the end, the goal is viral suppression, and both methods are achieving that. So, the patient should choose their treatment option. |
|  | Need for Pilot and Phased Roll-out | **H14:** I think it should first be launched at a small level in a specific area, to see patient compliance, the chances of success, and the issues involved. Once signs of success appear in one place, then it should be implemented at the national level.  **H17:** Then the second step for this would be then to do sort of mapping of the centres to see, would you do this for all the centres or only those which are eligible, which have the current feasibility to implement it. So, you don't want this in a really small centre in the middle of nowhere, which has no electric supply, instead a bigger centre in a larger city who has an electric supply, can get the medicine. And then finally talk about patients’ selection and then rolling out. |
|  | Digital and Health Information Systems for Monitoring | **H12:** A mobile service like in foreign countries could be helpful—where patients are contacted according to a predefined schedule. If that’s implemented, it would be a great improvement.  **H8:** See, there is no doubt, this possibility exists that the patient may forget or missed appointments can increase. But Alhamdullilah our DHIS and MIS systems are so strong that it reflects missed appointments and scheduled appointments. No, only it is showing to us, it is showing to the facility [ART centre]. |
|  | Integration into Existing Healthcare Models | **H11:** So, we will have to modify the MMD according to the life of the injectable. DSD is in our pipeline, and all the routine process will be initiated through this. Program is standing on a strong footing Thank God.  **H17:** The second thing is to have the protocols in place, which would mean at first, having this in the national guidelines, which is not difficult because I am the one who usually makes national guidelines, but would also have all of the contents we talked about that if somebody misses their injectable, what to do, who are the patient selections for which this is required or not.  **H11:** We will have to keep option for oral pills open since there would be refusals. |
|  | Strengthening Counselling Systems | **H3**: As an adherence counsellor, there will be a greater role to be played, by physician and the counsellors. First of all, to shift, move and switch people on this [injectable] we have to do counselling, ongoing counselling. A person who is curious about this might one day come and say that the medicine [pills] was fine. So, we have to tell them, tell them its benefits. So, it will be a long going process of switching. |
|  | Provider Optimism and Preparedness | **H14:** In my opinion, this is a very good program, and such advancements should continue to happen so that patients continue to benefit.  **H15:** No, honestly, we are not ready at all. Firstly, we don’t have trained staff, which is a basic requirement for this kind of service. Secondly, we lack the necessary infrastructure. The essential components needed to implement this process simply aren’t available at our centre right now. So, without proper training and infrastructure, we are not in a position to begin implementing this program. |
